# Grafted human-induced pluripotent stem cells-derived oligodendrocyte progenitor cells combined with human umbilical vein endothelial cells contribute to functional recovery following spinal cord injury

**DOI:** 10.1186/s13287-024-03651-1

**Published:** 2024-02-07

**Authors:** Qian Li, Sumei Liu, Tianqi Zheng, Mo Li, Boling Qi, Liping Zhou, Bochao Liu, Dan Ma, Chao Zhao, Zhiguo Chen

**Affiliations:** 1 Cell Therapy Center, Beijing Institute of Geriatrics, Xuanwu Hospital Capital Medical University, National Clinical Research Center for Geriatric Diseases, and Key Laboratory of Neurodegenerative Diseases, Ministry of Education, Beijing, China; 2 Center of Neural Injury and Repair, Beijing Institute for Brain Disorders, Beijing, 100069 China; 3 Center of Parkinson’s Disease, Beijing Institute for Brain Disorders, Beijing, 100069 China; 4 Translational Medicine Research Group (TMRG), Aston Medical School, Aston University, Birmingham, B4 7ET UK; 5 Department of Clinical Neurosciences, Wellcome Trust-Medical Research Council Stem Cell Institute, University of Cambridge, Cambridge, UK

**Keywords:** Spinal cord injury, Induced pluripotent stem cells, Oligodendrocyte progenitor cells, HUVECs

## Abstract

**Background:**

Spinal cord injury (SCI) is a devastating disease that causes extensive damage to oligodendrocytes and neurons leading to demyelination and axonal degeneration. In this study, we co-transplanted cell grafts containing oligodendrocyte progenitor cells (OPCs) derived from human-induced pluripotent stem cells (iPSCs) combined with human umbilical vein endothelial cells (HUVECs), which were reported to promote OPCs survival and migration, into rat contusion models to promote functional recovery after SCI.

**Methods:**

OPCs were derived from iPSCs and identified by immunofluorescence at different time points. Functional assays in vitro were performed to evaluate the effect of HUVECs on the proliferation, migration, and survival of OPCs by co-culture and migration assay, as well as on the neuronal axonal growth. A combination of OPCs and HUVECs was transplanted into the rat contusive model. Upon 8 weeks, immunofluorescence staining was performed to test the safety of transplanted cells and to observe the neuronal repairment, myelination, and neural circuit reconstruction at the injured area; also, the functional recovery was assessed by Basso, Beattie, and Bresnahan open-field scale, Ladder climb, SEP, and MEP. Furthermore, the effect of HUVECs on grafts was also determined in vivo.

**Results:**

Data showed that HUVECs promote the proliferation, migration, and survival of OPCs both in vitro and in vivo. Furthermore, 8 weeks upon engraftment, the rats with OPCs and HUVECs co-transplantation noticeably facilitated remyelination, enhanced functional connection between the grafts and the host and promoted functional recovery. In addition, compared with the OPCs-alone transplantation, the co-transplantation generated more sensory neurons at the lesion border and significantly improved the sensory functional recovery.

**Conclusions:**

Our study demonstrates that transplantation of OPCs combined with HUVECs significantly enhances both motor and sensory functional recovery after SCI. No significance was observed between OPCs combined with HUVECs group and OPCs-alone group in motor function recovery, while the sensory function recovery was significantly promoted in OPCs combined with HUVECs groups compared with the other two groups. These findings provide novel insights into the field of SCI research.

**Supplementary Information:**

The online version contains supplementary material available at 10.1186/s13287-024-03651-1.

## Background

Spinal cord injury (SCI) is a highly disabling disease of the central nervous system that causes severe sensory and/or motor deficits. Each year, between 250,000 and 500,000 people worldwide suffer from SCI, with approximately 90% of cases due to traumatic causes [1]. Traumatic SCI could be pathophysiologically divided into primary and secondary injuries [2, 3]. The primary injury produces immediate mechanical disruption, which causes massive necrosis of neurons, oligodendrocytes, and vasculature destruction [3–6]. Secondary injuries occur within several minutes of primary injury that could last for weeks to months [3, 7], which caused by cell permeabilization, pro-apoptotic signaling, and ischemic injury [2], resulting in the imbalanced microenvironment at the injured site. The consequent neuronal axonal rupture, demyelination [4–6], and formation of glial scars inhibit neuron and oligodendrocyte (OL) migration and axon extension [8], causing a severe deficit of motor and/or sensory functions. Studies using the rat spinal contusion injury reveal that the quantity of OLs decline within 15 min post-injury and extensive death occurs in the first 2 weeks. In particular, OLs apoptosis lasts for at least 3 weeks, leading to axon demyelination and degeneration [4, 9]. As a result, the Oligodendrocyte progenitor cells (OPCs) are activated and respond rapidly to SCI [8], as well as neuron progenitor cells. The maximum proliferation rate of OPCs appears at around 2 weeks post-injury [9], and differentiates into OLs to form myelin in 2–4 weeks after injury [10]. However, spontaneous remyelination is insufficient against OLs loss and demyelination; also it diminishes over time. Thus, replenishing exogenous OPCs while introducing another cell that could reduce the apoptosis of endogenous OPCs might promote remyelination after SCI.

Oligodendrocytes play a crucial role in neuronal support and signal transmission in the central nervous system (CNS). Studies have shown that OLs could provide energy support for neurons by participating in the pyruvate cycle and ATP synthesis, while neuron-glial antigen 2-expressing OPCs (NG2-OPCs) are involved in glutamate signal transduction and homeostasis maintenance in the CNS [11–14]. OLs wrap neuronal axons to form myelin sheaths to ensure rapid and saltatory conduction, demyelination may lead to reduced conduction velocity or conduction failure [15]. Studies have reported that myelinating oligodendrocytes are critical for the integrity of axonal structures and maintaining a "non-inflammatory" environment [16, 17]. Additionally, OPCs can form synapses with neurons and might differentiate into astrocytes or neurons under specific conditions [18–20]. Therefore, OLs loss would cause demyelination, which leads to the failure of energy-efficient conduction and the supportive role to axons and further leads to energy deficiency, perturbed axonal transport, and ultimately axonal degeneration.

Patients suffering from SCI need appropriate timely surgical and critical care, part of the neurological function might be regained, but most patients with severe injury still have irreversible neurological impairment. One of the most promising therapeutic strategies for SCI is exogenous cell therapy. In the past decades, studies have shown the feasibility and safety of cell transplantation into the injured spinal cord and improved functional recovery. Several sources of cells have been investigated in SCI treatment, neural stem cells (NSCs)/neural progenitor cells (NPCs) are capable of oligodendroglia differentiation in vivo [21, 22], but the majority of cells presented an astrocyte fate or remained as nestin-positive cells [22, 23]. Therefore, cell transplantation focused on oligodendrocyte fate may be more effective.

Endothelial cells play an important role in maintaining the homeostasis of the neuronal microenvironment through secreting VEGF, FGF-2, IGF-1, PDGF, BDNF, and other beneficial cytokines [24–28]. Many studies have demonstrated the crucial effects of endothelial cells on promoting axonal growth of dorsal root ganglia [27, 28], reducing apoptosis of OPCs [29], and promoting proliferation, migration, and remyelination of OLs [29–31]. In addition, endothelial cells can promote the survival and proliferation of NSC/NPC grafts [24]. HUVECs have been used as a more physiologically representative model for large vessel endothelial cells [28, 32, 33]. In the present study, we explored the roles of iPSCs-derived OPCs combined with HUVECs to provide functional benefits following transplantation in acute SCI.

## Methods

### Differentiation of OPCs from human iPSCs

The human iPSCs were reprogrammed from dermal fibroblasts (purchased from Pricella company, CP-H183) according to the protocol described by Yamanaka et al. [34]. hiPSCs infected with lentiviral green fluorescent protein (GFP) were cultured in E8 medium (STEMCELL 05990) and differentiated into oligodendrocyte progenitor cells according to the published protocol [35]. RA, SAG, SB431542, and LDN193189 were used to generate OLIG2^+^ progenitors. The key factors such as platelet-derived growth factor (PDGF), neurotrophin 3 (NT3), triiodo-l-thyronine (T3), insulin-like growth factor 1 (IGF-1), and hepatocyte growth factor (HGF) were used to promote the differentiation and/or survival of OPCs and oligodendrocytes. For the first 12 days, hiPSCs were seeded in adherent cultures and formed 3D structures by day 12. Then the adherent cultures were dissociated to form spheres in suspension to day 30. At this time point, spheres were plated into poly-D-lysine/laminin-coated dishes for migration and spread of the cells. PAX6 and OLIG2 were detected on day 8 and day 12 of differentiation, respectively. O4^+^/MBP^+^ OPCs were detected firstly on day 45, and the proportion gradually increased thereafter until day 75. Briefly, iPSCs were dissociated into single cells by using EDTA treatment at 37 °C for 5 min. Then the cells were seeded onto Matrigel at a low density (8000–10000 cells per cm^2^) in E8 medium supplemented with ROCK inhibitor (Gene Operation, Y27632). Cells were cultured in E8 medium to the next day, when the iPSC colonies reach a diameter of 100–250 μm in diameter, then the medium was replaced with neural induction medium. Differentiation was carried out from day 0 to day 8 using neural induction medium containing 100 nM RA (Sigma-Aldrich, cat. no. R2625), 10 µM SB431542 (Stemgent, cat. no. 04-0010), and 250 nM LDN193189 (Stemgent, cat. no. 04-0074) freshly added daily. From day 8 to day 12, N2 medium was used and added daily, which included 1 µM SAG (small-molecule smoothened agonist) and freshly prepared 100 nM RA. The adherent cells were mechanically dissociated into small clumps in suspension to form spheres on day 12. The clumps were transferred into an ultra-low-attachment 6-well plate, and two-thirds of the old medium was replenished every other day with fresh N2B27 medium until day 20. 100 nM RA and 1 µM SAG were added freshly on the day of use. From day 20 to day 30, the cell aggregates were cultured in PDGF medium that was the N2B27 medium including PDGF, HGF, IGF-1, and NT3, and two-thirds of the medium was aspirated and replenished with fresh medium every other day until day 30. On day 30, aggregates were plated in a 6-well plate coated with Poly-D-Lysine/laminin in a Glial induction medium including insulin, T3, biotin, cAMP, ascorbic acid (AA), and two-thirds of the medium was replenished every other day until day 85 of differentiation. Three independent differentiation experiments were performed, and each experiment included at least 3 technical replicates.

### HUVEC culture

HUVECs were purchased from iCell company (iCell–h110), and 1 × 10^6^ cells were plated in a T75 flask with 10 mL HUVEC complete growth medium (iCell–h110–001b), and the medium was refreshed every 2–3 days. When the cells reach 80–90% confluency, remove the medium and gently wash the cell with DPBS, digest cells into single cells by adding 3 mL 0.25% Trypsin–EDTA, and then reseed cells at a ratio of 1:4 to 1:5.

### OPC proliferation assay

OPC spheres were cultured with PDGF medium and HUVEC conditioned medium (CM) during day 20 to day 30 of differentiation. HUVEC-CM was used only in this section. HUVEC-CM was harvested when cells reached 70–90% confluency and sterilized by filtering through the 0.22 μm membrane. Around 10 spheres were placed in one well of an ultra-low-attachment 24-well plate. Cells were cultured with 1 mL PDGF medium and 300 µL HUVEC-CM, and two-thirds of the old medium was replenished every other day with fresh PDGF medium and HUVEC-CM until day 30. On day 30, the number of OPC spheres in each group was compared. Three independent experiments were performed in this section; each experiment was conducted with a minimum of three technical replicates.

### Transwell migration assay

On day 65, OPCs were isolated by O4-specific magnetic-activated cell sorting (MACS; Miltenyi Biotec) for transwell migration assay. Plated 3.5 × 10^4^ sorted and unsorted OPCs, respectively, into the inserts with a pore size of 8.0 µm (Millipore millicell hanging cell culture insert, MCEP24H48), and 7 × 10^4^ HUVECs into the underneath wells in the 24-well plate as shown. After 4 h of incubation, the membranes of the transwell insert were fixed in 4% PFA for 10 min, followed by DAPI nuclear counterstaining. Then, the migrated cells are counted for comparison. Three independent migration experiments were performed, and each experiment consisted of three technical replicates.

### Animals

A total of 40 female nude rats (8-week-old, around 180–200 g) were used in this study, animals were purchased from Vital River (China) and housed in National Institute for Occupational Health and Poison Control. Female rats are more docile, and have a lower chance of urinary tract infections post-injury compared to male rats, which simplifies the post-injury care required for injured female rats. Animals were maintained under a 12-h light/dark cycle, constant room temperature, and with free access to food and water ad libitum throughout the study. They were randomly divided by using random number tables method into four groups: control (*n *= 10), the rats transplanted with OPCs alone were referred to as the OPCs-alone group (*n *= 10), those transplanted with OPCs combined with HUVECs were referred to as the co-transplantation group (*n *= 14), and HUVECs-alone group represented the rats transplanted with HUVECs only (*n *= 6). The sample size was determined based on previous studies [36, 37]. We employed G*Power (version 3.1.9.2) software to calculate the statistical power, revealing a power of 0.797 based on our current sample size. Each animal was assigned a unique identification number, and detailed information was carefully recorded. This practice ensures accurate tracking and monitoring of individual animals throughout the study. Chinese Ministry of Public Health Guidelines and US NIH guidelines for laboratory animal care and safety were strictly followed. The animal experimental protocols were approved by the Laboratory Animal Ethics Committee of Xuanwu Hospital Capital Medicine University (Approval number: XW-20210423-2).

### Spinal cord injury

A contusion injury at T9–T10 was conducted to establish the spinal cord injury model. All surgery was done under deep anesthesia using 1% pentobarbitone (50 mg/kg). Anesthetized rats were given an around 2 cm incision at the midline skin along the T8–T11 thoracic vertebrae of the spinal cord. The paravertebral muscles were dissected bilaterally to visualize the spine, and laminectomy at the ninth and tenth thoracic spinal vertebrae was performed. The dorsal surface of the dura mater was exposed, and a contusion injury was induced by using an SCI device (PCI 3000; Hatteras, USA). The parameter settings were as followed: impactor tip size, 1.5 mm; impact velocity, 1.5 m/s; impact depth, 1.7 mm; and impact dwell time, 85 ms [38]. Following SCI, animals received cell transplantation. After transplantation, the muscle and skin were sutured, and the rats were given an intraperitoneal injection of penicillin (50,000 U/kg/day) consecutively for 5–7 days. Animals underwent manual bladder evacuation once a day until voluntary urine expression returned.

### Cell transplantation

OPCs were digested with prewarmed Accutase and resuspended in Glial medium; HUVECs were harvested and resuspended with OPCs at a ratio of 1:2, and cells were kept on ice until transplantation. Animals from different groups received the corresponding engraftment of Glial medium (control), OPCs, or OPCs combined with HUVECs. Cell suspensions or vehicle was injected with a 10 μl Hamilton Syringe connected with a 23-gauge needle in 5 sites at a 1.7 mm depth, including one injection at the injury epicenter, and four injections around its perimeter. For each rat, 1.5 × 10^6^ OPCs and 3 × 10^6^ HUVECs in a total volume of 15 µL vehicle were injected at a rate of approximately 2 µL/min, the needle was then kept in situ for an extra two minutes to prevent the extravasation of the injection.

### Functional test

The Basso, Beattie and Bresnahan (BBB) open-field locomotion rating scale [39] was assessed weekly, and the ladder beam walking task was assessed weekly during the first month following SCI and every other weekly in the second month. All testing was performed by two independent observers blinded to group identity. During weekly behavioral testing, the well-being of all animals was monitored and assessed. Within 3 days after SCI surgery, animals exhibiting outliers in BBB examination (with a value beyond mean ± 3 times SD), which indicates the failure of SCI modeling, would be excluded from further experiments and analysis.

### MEP and SEP test

Electrophysiological study was conducted for each group at week 8 following SCI (*n *= 7 rats/group, except for HUVECs group). Before the examination, the animals were anesthetized with 1% pentobarbital (50 mg/kg). Motor evoked potentials (MEP) and sensory evoked potentials (SEP) were measured with Keypoint-II bi-channel evoked potentials/electromyography.

For MEP, the stimulating electrode (needle electrodes) was placed under the scalp behind the ear and attached to the skull. The reference electrode was placed 1 mm beside the stimulation electrode. The recording electrode was inserted into the contralateral gastrocnemius muscle, 1 mm apart inserted the reference electrode, and the ground electrode was placed subcutaneously in the back of the rat.

For SEP, the stimulation electrode was inserted in the posterior tibial nerve of the hindlimb. The reference electrode was placed 1 mm beside the stimulation electrode. The recording electrode was placed subcutaneously on the gastrocnemius head, 1 mm apart inserted the reference electrode, and the ground electrode was placed subcutaneously in the back of the rat.

### Tissue processing

Animals were sacrificed at 2 weeks (*n *= 3 in HUVECs-alone group, *n *= 4 in co-transplantation group), 4 weeks (*n *= 3 in HUVECs-alone group), and 8 weeks (*n *= 10 in control group, OPCs-alone and co-transplantation groups) after transplantation. The animals were weighed and subsequently received an intraperitoneal injection of 1% pentobarbital (50 mg/kg). After confirming that the animals had reached a state of deep anesthesia, indicated by the absence of the corneal reflex and relaxation of the limb muscles, arterial perfusion with 0.9% saline and 4% paraformaldehyde (PFA) was performed sequentially. The spinal cords were removed and post-fixed in 4% PFA overnight at 4 °C, and cryoprotected in 30% sucrose in PBS for 48–72 h at 4 °C. The tissues were embedded in Optimal cutting temperature (O.C.T) compound and stored at -80 °C until sectioning at 20 μm thickness on a Cryo-Ultramicrotome (Leica). All animal tissue sections were sagittal serial sections.

### Immunohistochemistry

For cell immunofluorescence staining, the cells were plated onto Poly-D-Lysine/Laminin-coated coverslips at different time points during differentiation. The cells were fixed by applying ice-cold 4% paraformaldehyde (PFA) for 10 min at RT, followed by three washes with PBS. The cultures were blocked with 3% normal donkey serum in PBS/PBST for 60 min, followed by incubation with primary antibodies (the list of antibodies was shown in Additional file 1: Table S1) at 4 °C overnight. The conjugated secondary antibodies (Immune-Jackson, Inc., CA, USA) were incubated on the next day for 2 h at RT.

For tissue sections, the sections were washed with PBS, blocked for 90 min with 3% normal donkey serum in PBST, and incubated with primary antibodies at 4 °C overnight. The next day, the sections were washed with PBST and incubated with conjugated secondary antibodies for 2 h at RT. DAPI was added for nuclear counterstaining in the end.

### Statistical methods

Data were expressed as mean ± SEM and analyzed using GraphPad Prism 9 (GraphPad Software, La Jolla, CA). The length of axon growth in vitro and in vivo, the lesion area, demyelination area, and immunofluorescence signal abundance were measured by using ImageJ software (version 1.52 k). For each animal, every 10th tissue sections containing the injury site and grafts were assessed. Measurement of axon growth of grafted cells in vivo was performed by immunofluorescence staining of spinal cord tissue sections, and the distance of axonal extension from the injection site to the rostral and caudal of spinal cord was measured; the longest distance of axonal extension of grafts was presented. Migration assay, proliferation experiment, and axon growth length comparison between two groups were assessed using Student’s t test.

BBB scores and Ladder beam scores were analyzed by repeated measures two-way ANOVA with Tukey’s multiple comparison test at each time point. Based on the BBB score, we exclude several animals (*n *< 3) for the following analysis. MEP and SEP test, for comparison of cell types proportion, calretinin-positive cells proportion, the lesion area, demyelination area, and immunofluorescence signal abundance, was analyzed by repeated measures one-way ANOVA with Tukey’s multiple comparison test in different groups. The differences were set as statistically significant when *p* value was less than 0.05.

## Results

### *hiPSCs successfully differentiate into mature MBP* + *oligodendrocytes *in vitro

**Fig. 1:**
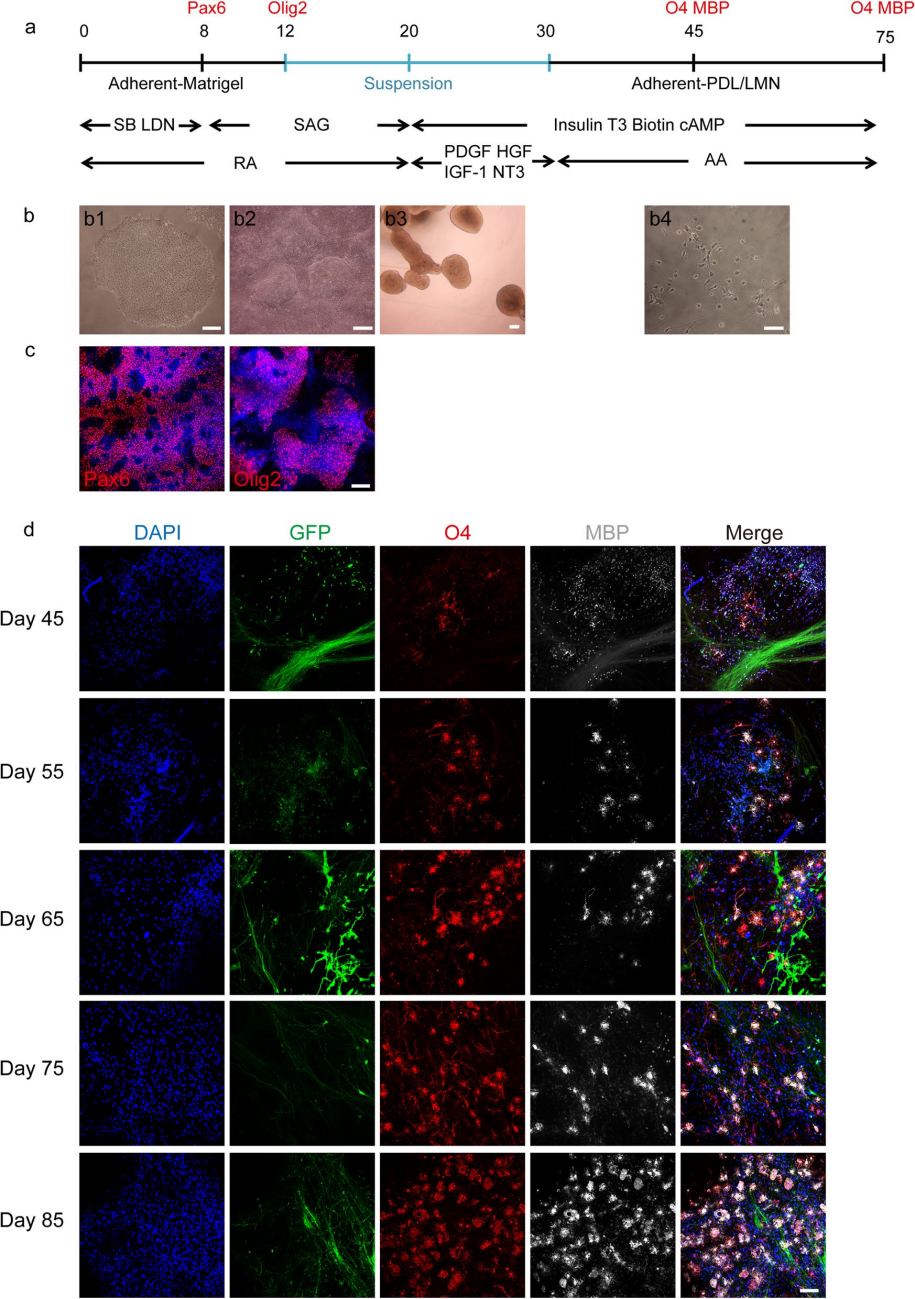
Scheme of hiPSC differentiation to oligodendrocytes. **a** Timeline of oligodendrocyte induction. **b** Morphology of the culture at different stages of differentiation from iPSCs to oligodendrocytes. Preparation of hiPSC cultures for differentiation (b1), cells were induced into OLIG2^+^ progenitors at day 12 (b2), then mechanically dissociated the cultures to form spheres during day 20 to day 30 (b3). After 45 days of differentiation, OPCs migrated out of the sphere and matured (b4). Scale bars, 100 μm. **c** Expression of PAX6 and OLIG2 at day 8 and day 12, as shown through immunofluorescence analysis (PAX6/OLIG2, red; GFP green; DAPI, blue). Scale bars, 100 μm. **d** O4^+^ and MBP^+^ stainings during the differentiation process. The proportion of O4^+^ and MBP^+^ OPCs gradually increased from 45 to 85 days (O4, red; MBP, white; DAPI, blue). Scale bars, 100 μm

**Fig. 2:**
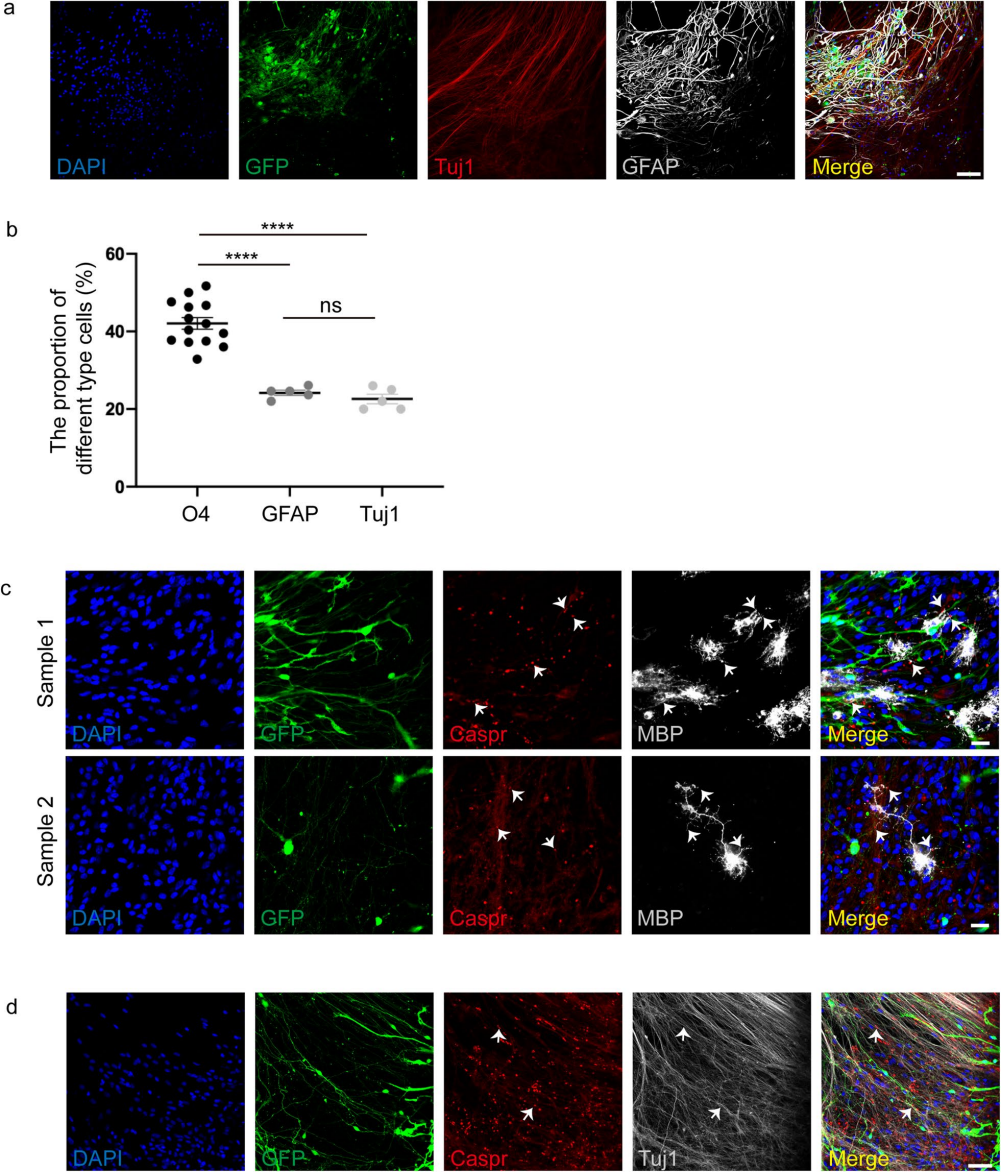
Oligodendrocytes form Nodes of Ranvier in vitro. **a** Expression of Tuj1 and GFAP at day 85 (GFP, green; Tuj1, red; GFAP, white). Scale bars, 100 μm. **b** Different cell types proportion generated on day 75 of differentiation. Data are means ± SEM, ns, no significance; *p < 0.05, **p < 0.01, ***p < 0.001, ****p < 0.0001, One-way ANOVA, *N *= 3 independent experiments. **c**–**d** Immunofluorescence staining of Caspr/MBP and Caspr/Tuj1 at day 75. Nodes of Ranvier (arrowheads) were co-labeled with MBP and Tuj1 (Caspr, red; MBP/Tuj1, white). Scale bars, 25 μm (**c**), 50 μm (**d**)

hiPSCs were induced into OPCs according to published protocols(Fig. 1a) [35]. During the process of differentiation, cell morphology at various stages is shown in Fig. 1b. PAX6 and OLIG2 were detected on day 8 and day 12 of differentiation, respectively, indicating that hiPSCs were induced into OLIG2^+^ progenitors (Fig. 1c). From this time point, cells were dissociated to form spheres to enriched OLIG2^+^ population. Subsequently, starting from day 30 of differentiation process, OPCs were driven to mature OLs. In our study, O4^+^/MBP^+^ OPCs were detected firstly on day 45, and the proportion gradually increased thereafter until day 75 (Fig. 1d). The cultures also showed the presence of GFAP^+^ astrocytes and Tuj1^+^ neurons (Fig. 2a). We identified variable proportions of other cell types generated during the differentiation on day 75: O4^+^ oligodendrocytes were about 42% ± 6%, GFAP^+^ astrocytes were about 24% ± 2%, and Tuj1^+^ neurons were 23% ± 3% (Fig. 2b). Furthermore, we successfully induced OPCs from other stem cell lines: iPSC, hESC (line WA09 [WiCell], passages 20–40), and hESC-EGFP gifted from Professor Yuejun Chen (Additional file 1: Fig. S1a), suggesting our differentiation procedure was reliable.

At day 35–39 of differentiation, we transplanted hiPSC-GFP-OPCs into a nude rat SCI model. We identified different cell types before transplantation in vitro: the day 35–39 cultures were characterized for cellular composition which included about 29.6% ± 3% of O4-positive cells, about 24.4% ± 2% of PDGFRα-positive cells, and 21.1% ± 2% of Tuj1-positive cells (Additional file 1: Fig. S1b, c).

In the CNS, OLs can wrap neuronal axons and form myelin sheaths, and the unmyelinated portions present between myelinated segments of the axons are named nodes of Ranvier, to ensure rapid and saltatory conduction of action potentials [15]. In our study, we observed MBP^+^ cells expressed Caspr (contactin-associated protein), a marker of Ranvier nodes [40], and Caspr was co-labeled with Tuj1 on day 75 of differentiation (Fig. 2c, d), indicating the OLs induced by OPCs matured.

### *HUVECs promote OPC proliferation, migration, survival, and neuronal axonal growth *in vitro

**Fig. 3:**
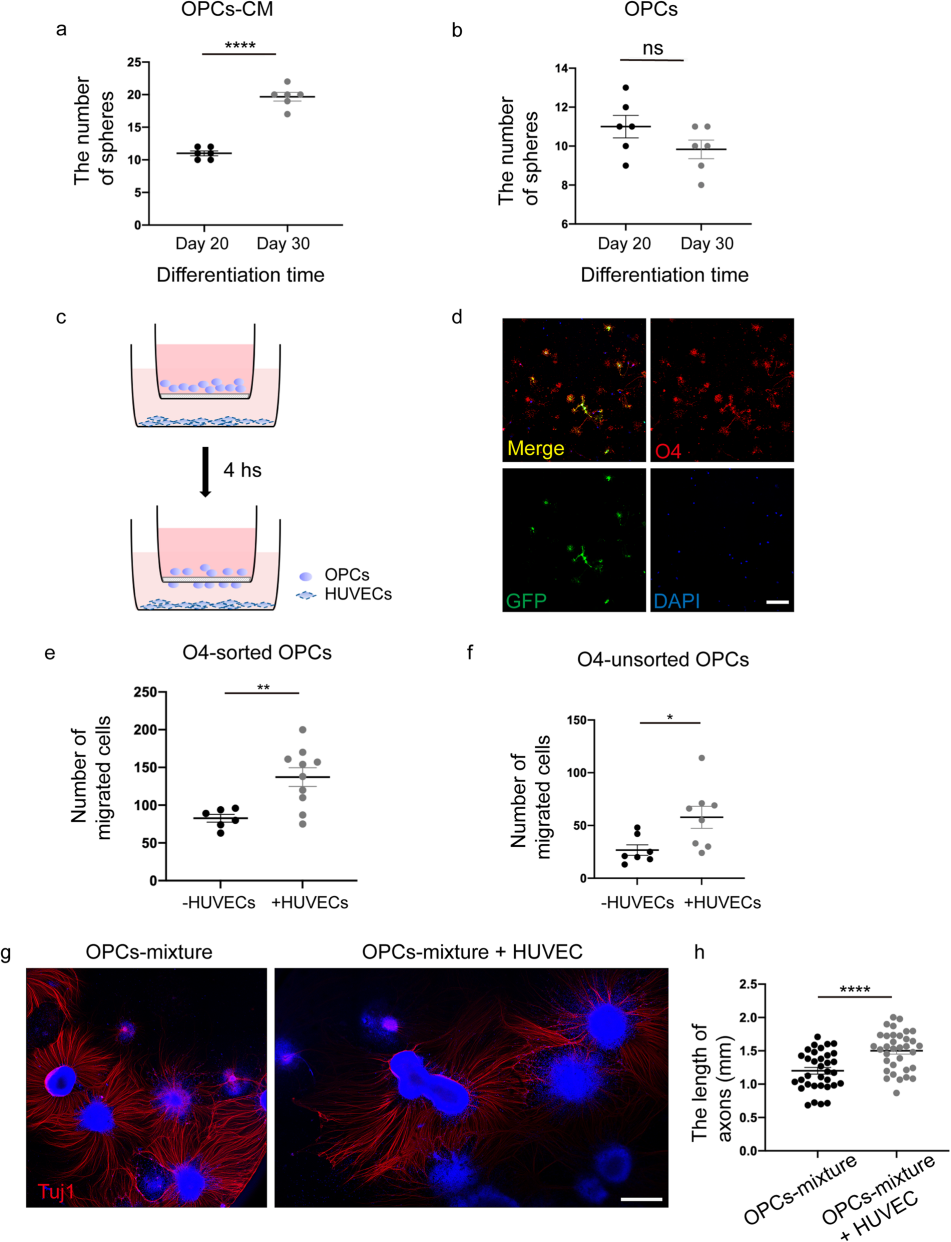
HUVECs promote OPCs proliferation, migration, and neuronal axonal growth in vitro. **a–b** The number of spheres in the CM-culture group (**a**) significant increase compared with the control group (**b**) during day 20 to day 30 of differentiation. Data are means ± SEM, ns, no significance; *p* < 0.05, **p < 0.01, ***p < 0.001, ****p < 0.0001, t test, *N *= 3 independent experiments. **c** Schematic diagram of migration experiment. **d** Immunofluorescence staining of the sorted OPCs (O4, red; GFP, green). Scale bars, 100 μm. **e**–**f** The number of migrated O4 sorted (**e**) and unsorted (**f**) cells was increased when co-cultured with HUEVCs for 4 h. Data are means ± SEM. *p < 0.05, **p < 0.01, t test, *N *= 3 independent experiments. **g** Axon extension of neurons in the OPCs mixtures cultured with/without HUVECs after three days (Tuj1, red; DAPI, blue). Scale bars, 750 μm. **h** Compared with the OPCs-mixture group, the axon length of neurons in the OPCs-mixture + HUVEC group was significantly increased. Data are means ± SEM. *p < 0.05, **p < 0.01, ***p < 0.001, ****p < 0.0001, t test, *N *= 3 independent experiments

In the current study, the number of OPC spheres which mainly contain OLIG2-positive cells remained consistent on day 20 versus on day 30 of differentiation; with the addition of HUVEC conditioned medium (CM), we noticed a significantly increased number of spheres compared with the culture without CM (Fig. 3a, b). On day 65, 3.5 × 10^4^ O4^+^ sorted and unsorted OPCs, respectively, were plated into the inserts, and 7 × 10^4^ HUVECs into the underneath wells in the 24-well plate for transwell migration assay (Fig. 3c). To verify the purity of sorted OPCs, cells were identified after 3-days culture (Fig. 3d). Compared with the control group, the migrated cells were significantly increased in the HUVECs group after 4 h of incubation (Fig. 3e, f, Additional file 1: Fig. S2a). Furthermore, to assess the effect of HUVECs on the growth of neuronal axons, 8–10 OPC spheres were co-cultured with 5 × 10^3^ HUVECs at day 30. The length of axons as revealed by Tuj1 staining was found to be significantly longer in the co-culture group with HUVECs than the control group without HUVECs on day 3 (Fig. 3g, h). Similar results were obtained following the co-culture of ESC-derived OPCs with HUVECs (Additional file 1: Fig. S2b, S2c). On day 45, OPCs were digested and reseeded with or without HUVECs (5 × 10^3^/well of a 24-well plate) for 3 days. After incubation, the ability of axon extension, cell migration, and survival was found to be blocked without HUVECs (Additional file 1: Fig. S2d). Taken together, these data implied that HUVECs are beneficial to the proliferation, migration, survival of OPCs, and the axon growth of neurons.

### Survival and migration of transplanted hiPSC-GFP-OPCs in the SCI animal model

**Fig. 4:**
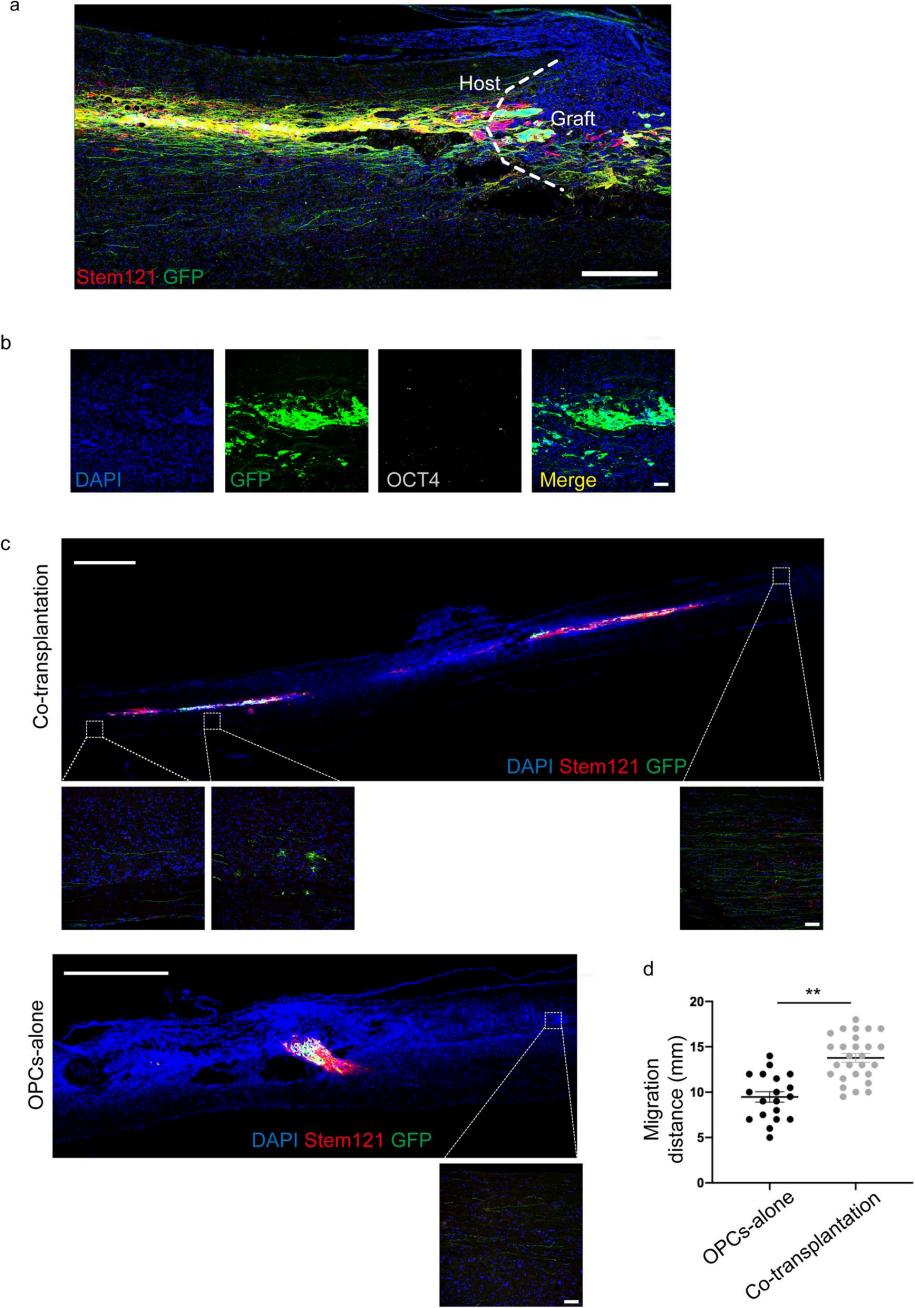
Survival, safety, and migration of engraftments in the SCI model 2 months post-transplantation. **a** Overview of GFP and Stem121 fluorescent immunolabeling demonstrates graft could survival, and migration 8 weeks post-grafting (Stem121, red; GFP, green). Scale bar, 500 μm. **b** OCT4 was negative in the transplanted cells (OCT4, white). Scale bar, 100 μm. **c** Grafts migrated to rostral and caudal of the host spinal cord, and the high-magnification view of the regions indicated by the inserts (Stem121, red; GFP, green). Scale bar, 2000 μm; high-magnification view, 100 μm. **d** The average migration distance of the co-transplantation group was 13.78 mm ± 0.48 mm, and the OPCs transplantation group was 9.47 mm ± 0.58 mm. The migration of the co-transplantation group was significantly enhanced (Stem121, red; GFP, green). Data are means ± SEM. *p < 0.05, **p < 0.01, t test, *n *= 5 rats in each group. The rats transplanted with OPCs alone were referred to as the OPCs-alone group, and those transplanted with OPCs combined with HUVECs were referred to as the co-transplantation group

Next, we transplanted hiPSC-GFP-OPCs at day 35–39 of differentiation with or without HUVECs into a nude rat SCI model. Cell viability was over 90% after passing through the Hamilton syringe needle by using Trypan blue staining. Cellular composition of the differentiated cells before transplantation was identified in vitro (Additional file 1: Fig. S1b, c). Eight weeks following transplantation, the SCI rats were euthanized and immunofluorescence staining was performed on the spinal cord sections to examine the safety, survival, and migration of the transplanted cells. The results showed that the transplanted OPCs could survive, cross the lesion site, and migrate away from the transplantation site in both groups, with or without HUVECs. The grafts were stained for Stem121, a marker of human cells, GFP, and OCT4 on the spinal cord sections 8 weeks following transplantation (Fig. 4a, b) to confirm the survival and safety of the grafts. HUVECs, which stained for PECAM-1, in the co-transplanted graft could not be detected two weeks following transplantation, while the OPCs could survive (Additional file 1: Fig. S3a, b). Similarly, HUVECs were not detectable in 4 weeks post-transplantation (data not shown). The grafts presented with a strong migratory ability and were found to cross the lesion site and migrate to the rostral and caudal parts away from the lesion core at the spinal cord (Fig. 4c, Additional file 1: Fig. S3c). The migration distance of grafts in the co-transplantation group was 13.78 mm ± 0.48 mm which was significantly longer than 9.47 mm ± 0.58 mm in the OPCs alone group, suggesting that HUVEC graft might have promoted the migration of transplanted cells in vivo (Fig. 4d). These results demonstrate that hiPSC-derived OPCs could survive and non-tumorigenesis in vivo, and HUVECs exhibited a beneficial effect on grafts migration.

### Grafted hiPSC-GFP-OPCs contribute to the integrity of the injury area, remyelination, and synapse formation

**Fig. 5:**
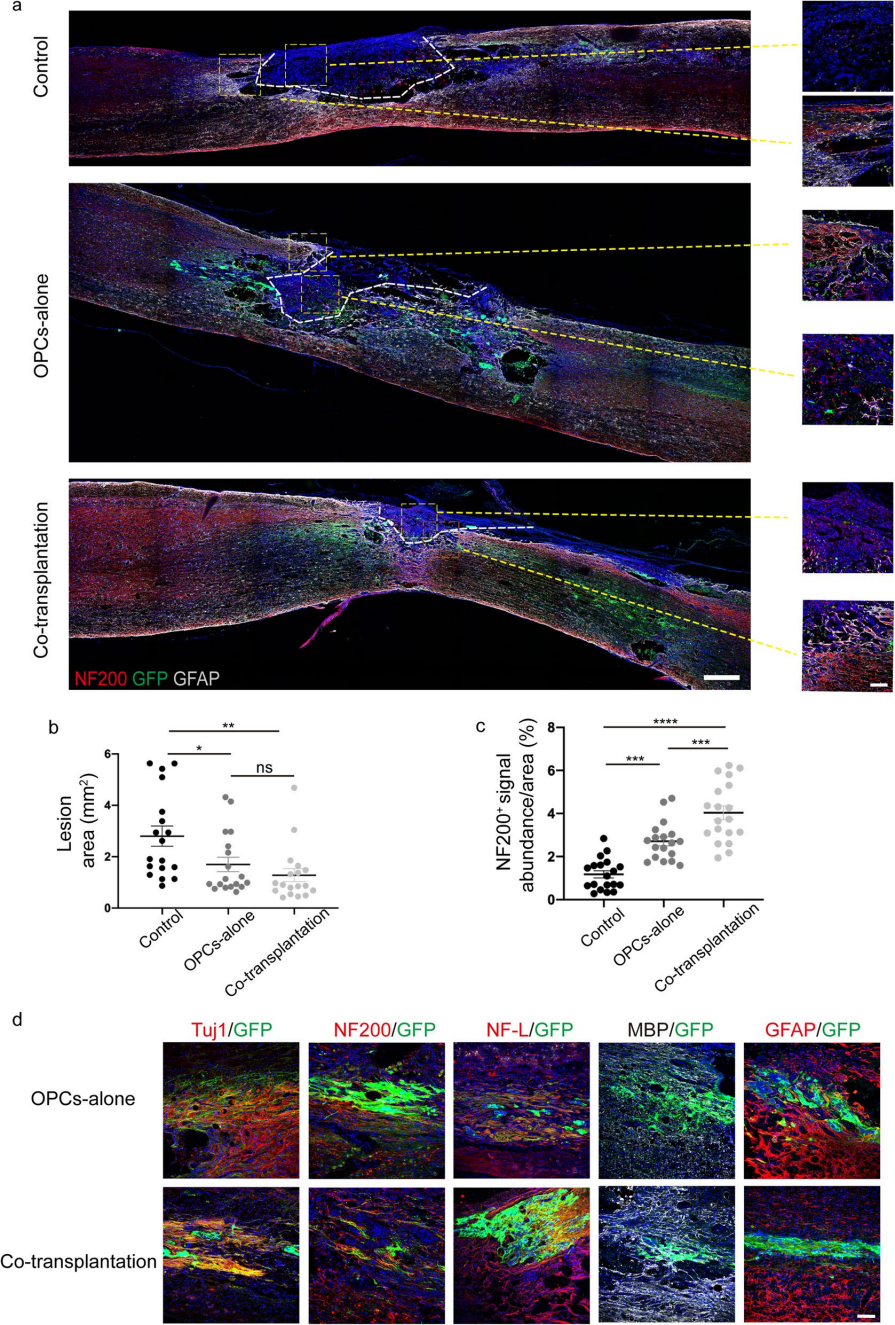
The tissue morphology, and grafts differentiation after 2 months of transplantation in vivo. **a** GFP^+^, NF200^+^ staining in the damaged area of each group after 8 weeks of transplantation. The high-magnification view of the regions was shown at the right side (GFP, green; NF200, red; GFAP, White). Scale bar, 500 μm (left panel), 75 μm (right panel). **b** Size of lesion areas in each group. The damaged area of transplantation groups was significantly decreased. Data are means ± SEM. *p < 0.05, **p < 0.01, One-way ANOVA, *n *= 6 rats in each group. Scale bar, 500 µm. **c** Compared with the NF200 pixel abundance in the lesion area of the control group, cell transplantation increased neurons density (NF200, red). Data are means ± SEM. *p < 0.05, **p < 0.01, ***p < 0.001, One-way ANOVA, *n *= 6 rats in each group. Scale bar, 75 µm. **d** Grafts differentiated into Tuj1/NF200/NF-L neurons, MBP oligodendrocytes, and GFAP astrocytes (GFP, green; Tuj1/NF200/NF-L/GFAP, red; MBP, white). *n *= 5. Scale bar, 100 μm

Eight weeks post-transplantation, we analyzed the histology of the identified injured area (within the dotted line) of the spinal cord in each group (Fig. 5a, Additional file 1: Fig. S3d). The total area of the lesioned areas in the transplantation groups was significantly smaller than that in the control group without engraftment (Fig. 5b). Moreover, the transplantation groups exhibited a notably greater abundance of NF200^+^ signals in the lesion area compared with the control group (Fig. 5c), with the co-transplantation group showing a higher NF200^+^ signal abundance than the OPCs-alone group. We also evaluated the differentiation capacity of the grafts following transplantation in vivo, and found that the engrafted cells could be further differentiated into Tuj1^+^ (37.3%) /NF200^+^ (31.6%) /NF-L^+^ (27.9%) neurons, MBP^+^ oligodendrocytes (43.5%), and GFAP^+^ astrocytes (15.4%) (Fig. 5d).

**Fig. 6:**
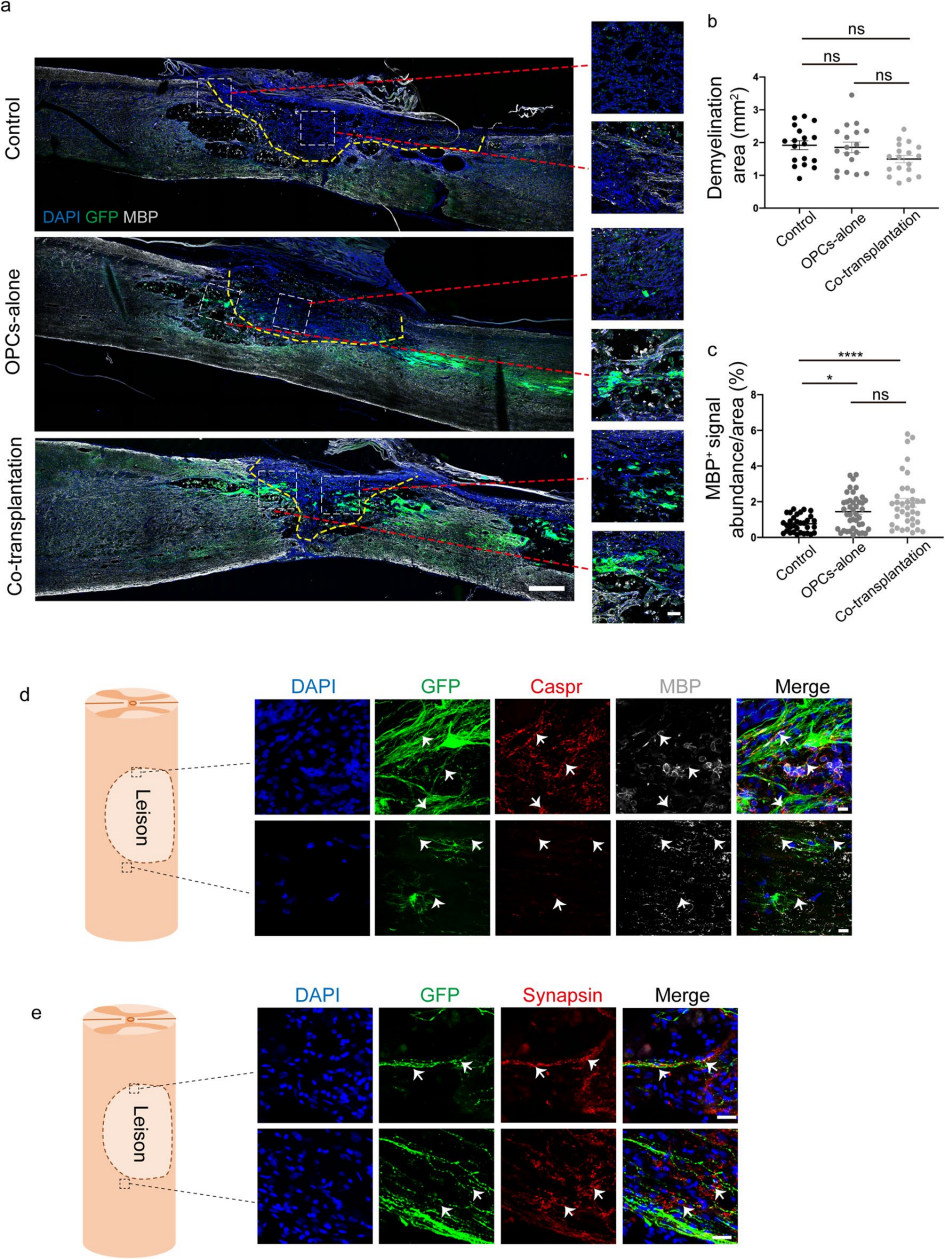
Grafts contribute to remyelination and synapse formation 2 months post-transplantation in vivo. **a** MBP immunofluorescence staining showed demyelination/remyelineation in each group post-injury. The demyelination area was more extensive in the control group and fewer MBP^+^ cells in the lesion site than in the transplantation group, the high-magnification view of the regions indicated by the white box (GFP, green; MBP, White). Scale bar, 500 μm (left panel), 75 μm (right panel). **b** Statistics of demyelination areas in each group. Data are means ± SEM, ns, no significance; one-way ANOVA, *n *= 6 rats in each group. **c** Compared with the MBP pixel abundance of the control group in the lesion site, grafts promoted remyelination (MBP, white). Data are means ± SEM, ns, no significance; *p < 0.05, **p < 0.01, One-way ANOVA, *n *= 6 rats in each group. **d** GFP was co-labeled with Caspr/MBP (arrowheads), indicating the grafts formed nodes of Ranvier in vivo (GFP, green; Caspr, red; MBP, white). Scale bar, 25 μm. **e** The grafts form synapse (arrowheads) in vivo by co-staining of GFP and Synapsin (GFP, green; Synapsin, red). Scale bar, 50 μm

The MBP^+^/GFP^+^ cells were detected at and around the lesion area, and the area of demyelination (within the dotted line), which was identified by MBP staining, in the control group seemed to be more extensive than that in the cell transplantation groups but the difference was not statistically significant (Fig. 6a, b), whereas the signal abundance of MBP^+^ cells at the lesion area was significantly greater in the engraftment groups than that in the control group (Fig. 6c), indicating that the grafted OPCs might differentiate into mature oligodendrocytes and promote remyelination. The nodes of Ranvier (Caspr^+^) co-labeled with MBP/GFP were observed in the transplantation groups, suggesting that the graft-derived mature OLs might have wrapped neuronal axons and formed myelin sheath (Fig. 6d). To access the ability of transplanted cells to integrate with host neuronal circuits, double immunostaining with antibodies against synapsin and GFP was performed. The results revealed the presence of synapsin signals that were co-localized with GFP (Fig. 6e), suggesting that the grafts could have integrated with host neuronal circuits and formed synapses.

### Grafts enhance functional recovery following SCI

**Fig. 7:**
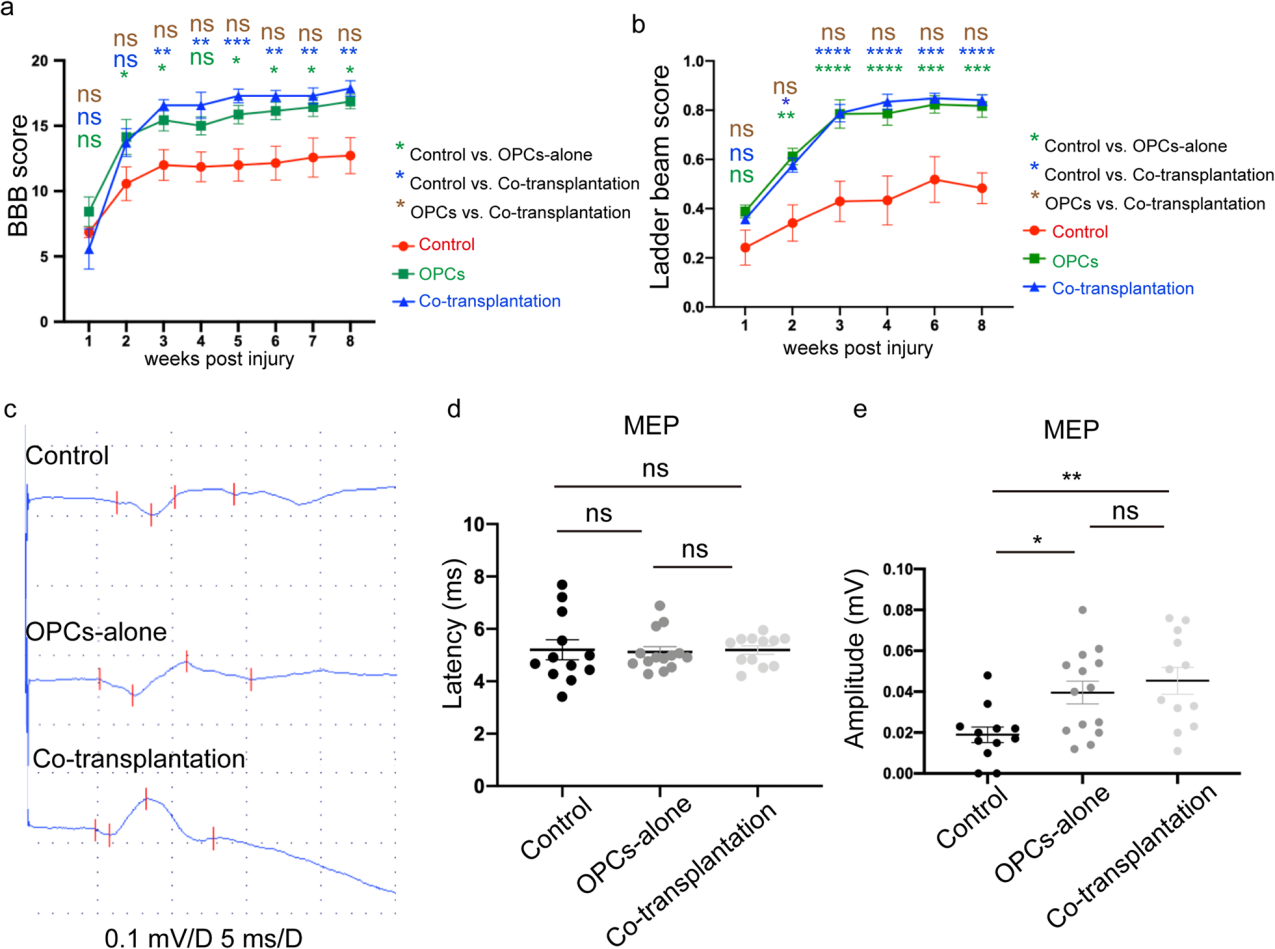
Significant motor functional improvement after T9 contusion injury**. a**–**b** BBB score (**a**) and Ladder climb score (**b**) in each group within eight weeks after SCI. Data showed behavior tests of grafted groups were significantly improved compared with the control group. Data are means ± SEM, ns, no significance; *p < 0.05, **p < 0.01, ***p < 0.001, **** *p *< 0.0001, Two-way ANOVA, *n *= 7 rats in each group. **c** Electrophysiology of all groups. **d**–**e** MEP latency showed no significance among the three groups (**d**), while the amplitude in the grafted groups was significantly higher than in the control group (**e**). Data are means ± SEM, ns, no significance; *p < 0.05, **p < 0.01, ***p < 0.001, one-way ANOVA, *n *= 6 rats in control group and co-transplantation group, *n *= 7 rats in OPCs-alone group

**Fig. 8:**
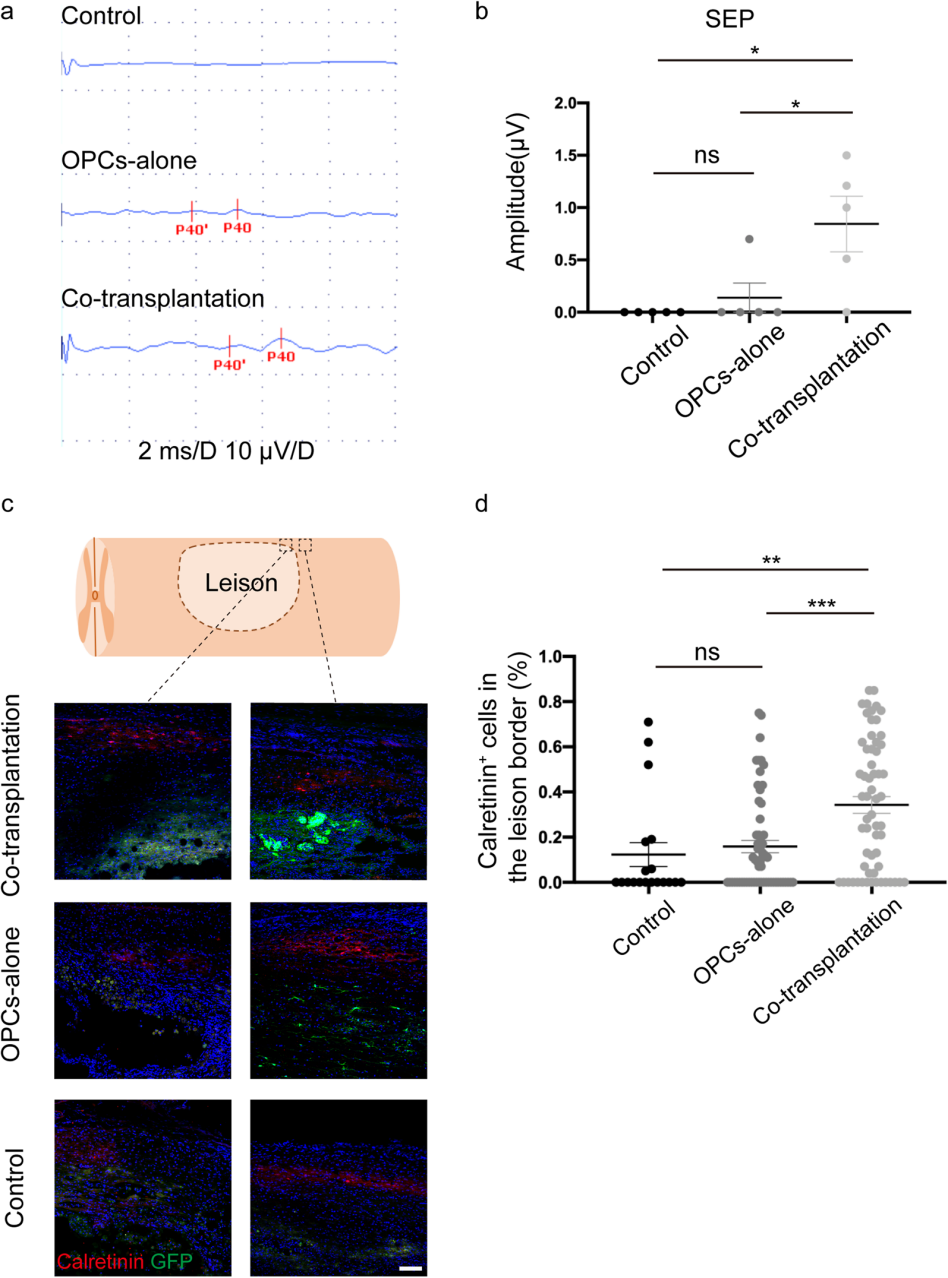
OPCs combined with HUVECs implant promote sensory function recovery after SCI. **a** Electrophysiology of all groups. **b** SEP amplitude was significantly higher in the co-transplantation group than in the OPCs transplantation group and the control group. Data are means ± SEM, ns, no significance; *p < 0.05, One-way ANOVA. *n *= 5 rats in each group. **c** Immunofluorescence staining of Calretinin in injury border in all groups. Scale bar, 100 μm. **d** Calretinin^+^ cell counts in injury border showed significant differences between the co-transplantation group with the OPCs transplantation group and the control group. Data are means ± SEM, ns, no significance; *p < 0.05, **p < 0.01, one-way ANOVA, *n *= 5 rats in each group

Functional recovery was assessed by using BBB open-field locomotor rating scale, Ladder beam scale, and MEP (motor evoked potential)/SEP (sensory evoked potential) tests. The BBB score and Ladder beam score indicated a noticeable improvement in motor function in the transplantation groups compared to the control group at 3 weeks post-SCI and thereafter beyond (Fig. 7a, b). We also examined the MEP/SEP at 8 weeks post-transplantation to validate the recovery of motor and sensory functions. MEP waves were detected in all groups (Fig. 7c); there were no significant differences in MEP latency among the three groups, but the MEP amplitudes were markedly higher in the transplantation groups in comparison to the control group (Fig. 7d, e). On the contrary, SEP waves were only detected in 80% (4 out of 5) of animals in the co-transplantation group, and 20% (1 out of 5) in the OPC-alone transplantation group but were absent in the control group, and the amplitudes were significantly higher in the co-transplantation group than those in the other two groups (Fig. 8a, b). Furthermore, the immunostaining results revealed a higher proportion of sensory neurons positive for calretinin in the injury border of the co-transplantation group than that in the OPC-alone transplantation group and the control group [41–43] (Fig. 8c, d). However, the calcitonin gene-related peptide (CGRP) signals, which labeled the axons of sensory neurons [44–48], were detected around the lesion area, the abundance of CGRP signals tended to increase in the transplanted groups, but there was not statistically different among the three groups (Additional file 1: Fig. S4a, S4b). The results suggested that co-transplantation of iPSC-derived OPCs culture together with HUVECs might have promoted the recovery of sensory functions in SCI rats.

## Discussion

In this study, we induced human iPSCs to differentiate into OPCs, which developed into mature OLs in vitro, and then transplanted OPCs with HUVECs after SCI in vivo. In the series of experiments, we found that HUVECs promote OPCs proliferation, migration, survival, and neuronal axonal growth both in vitro and in vivo. Moreover, OPCs-HUVECs grafts promoted motor and sensory function recovery after injury, suggesting good potential in SCI therapy.

Spinal cord injury would cause OLs death at the injury site directly which leads to primary demyelination, neuronal or axonal loss also leads to secondary demyelination [49]. OLs loss and axonal demyelination would further lead to the impairments of signal propagation, transportation of essential neurotrophic factors for neurons survival, and secondary axonal damage [50]. Therefore, transplanting exogenous OPCs could be advantageous for SCI recovery. However, oligodendrocytes are less abundant in vivo [8, 11], and difficult to obtain. Many researchers have been exploring the differentiation of hiPSCs and hESCs into oligodendrocytes. In 2009, Hu. et al. successfully differentiated hESCs into oligodendrocytes in a 16-week period, with a differentiation efficiency of O4^+^ cells of about 40% [51]. Subsequently, many successful protocols for differentiating hESCs/hiPSCs into oligodendrocytes were published, shortening the experimental period but without great improvement in the differentiation efficiency, and the efficiencies of O4^+^ cells obtained range from 4 to 47% [52–56]. Wang et al. developed a six-stage differentiation protocol, achieving a higher OPCs production of hiPSC than hESC, but still at the cost of a lengthy process [52]. Based on the previous work, Douvaras et al. combined dual SMAD inhibition with RA application, and successfully generated O4^+^ OPCs within 55 days, while maintaining a relatively high differentiation efficiency, with O4^+^ OPC at about 30%. This study significantly shortened the differentiation time of OPCs [35]. The successful protocol of inducing iPSCs into OPCs represents a significant advancement for OPCs transplantation after SCI. This is due to the fact that iPSCs can be derived from a patient's somatic cells, avoiding ethical issues and immune rejection reactions. The ability to generate patient-specific OPCs using iPSCs opens up new possibilities for personalized regenerative therapies for SCI.

In the present study, the differentiation protocol of Douvaras et al. was followed and slightly optimized. We observed O4^+^ OPCs on day 45 of differentiation, shortening the induction cycle of OPCs from iPSCs. This might be related to the aggregation of olig2^+^ cells at day 12, the cell layer in one well was dissociated into evenly sized 7 × 7 pieces in our study. In Douvaras et al. protocol, cell clumps are smaller than 20 × 20 pieces in one well. Therefore, cell clumps are larger and more accessible to form spheres, thus shortened the differentiation time. In addition, these differentiated OLs can form nodes of Ranvier in vitro. During this differentiation protocol, O4^+^ oligodendrocytes were generated about at 42% ± 6%. However, it is still hard to induce high-purity OPCs from iPSCs differentiation currently. Therefore, further research is required to explore the induction conditions of pure OPCs from other cells. Moreover, due to the poor viability of OPC, HUVECs were introduced as an assist cell to improve OPC survival.

Recent studies have shown endothelial cells have a positive effect on the proliferation, migration, and survival of OPCs, as well as the growth of axons. Masashi Kurachi et al. reported that conditioned medium (CM) of endothelial cells could inhibit the apoptosis of OPCs and promote their proliferation, migration, and survival in vitro [29], and the transplantation of brain microvascular endothelial cells (MVECs) into white matter infarcts in rats could reduce the apoptosis of OPCs and promote remyelination in the infarcted area [29]. Also, in vivo, migration of OPCs requires the presence of vasculature in the CNS as a physical substrate for traveling along the blood vessels or jumping between vessels [30]. HUVECs can also promote the growth of dorsal root ganglion axons by secretion of brain-derived neurotrophic factor (BDNF) [28]. A recent study conducted by Dai et al. shows that primary spinal cord-derived microvascular endothelial cells (SCMECs) promoted spinal cord or brain-derived NSC (SNSC/BNSC) proliferation, migration, and differentiation in vivo, and transplantation of SCMECs with the NeuroRegen scaffold into complete SCI rat model has beneficial effect on both vascular reconstruction and neural regeneration [57]. Moreover, stimulation of MSCs with exosomes (EX) derived from hypoxic preconditioned HUVEC efficiently enhances the angiogenic function of MSCs, and induces nerve recovery in the treatment of transection SCI models [58].

In our study, HUVECs conditioned medium (CM) was added to OPCs culture and showed that HUVECs-CM could promote the proliferation of OPCs. Endothelial cells can secrete multiple cytokines such as VEGF, IGF-1, PDGF, NT-3, BDNF, etc. [24–28]. But whether one or several factors jointly promote the proliferation, migration, and survival of OPCs or the direct contact with OPCs plays a facilitating role is unclear. Unfortunately, this study lacks explorations of the underlying mechanism of the promoting effect of HUVECs on OPCs. We plan to conduct in-depth research in future by acknowledging this deficiency. Additionally, the difference between indirect effects and direct contact of HUVECs in promoting the differentiation of OPCs is also worthy of further exploration.

In this study, unsorted OPCs were used for transplantation and were actually mixed cells, which contain OPCs, astrocytes, and neurons. This is because first, spinal cord injury can cause death and degeneration of neurons and glial cells, and damage to oligodendrocytes can lead to abnormal axonal energy metabolism and axonal degeneration of neurons [12, 59]; at the same time, neuronal damage also affects the survival of oligodendrocytes; astrocytes supply energy to neurons and also play essential roles in immunity, regulation of energy metabolism, and synaptogenesis[60–62]. Studies have found that the reduction of OPCs suppresses astrocytic development, astrocytic network formation and activity [63]. Moreover, the recruitment and remyelination of OPCs in inflammation areas also depend on astrocytes [64]. In this study, we found that purified O4^+^ cells will undergo apoptosis after 3–5 days when cultured alone in vitro. Therefore, transplantation of purified OPCs after SCI may not be conducive to its repair: on the one hand, it cannot replenish the cell types lost during the injury; on the other hand, the microenvironment of the lesion site will be less suitable for the survival of OPCs in the absence of neurons and astrocytes.

In vivo, studies revealed that the grafts contribute to remyelination and synapse formation. Motor functional recovery was significantly better in the implanted groups than in the control group, especially in the co-transplanted group. The BBB test and the ladder beam scores of the co-transplantation group were higher than the OPCs transplantation group, but there was no significant difference. Interestingly, the sensory function was improved in the co-transplanted group, but not in the OPCs-alone and control groups. We conducted the SEP test to assess the recovery of sensory functions after SCI. Data showed that SEP waves could be detected in about 80% (4 of 5) of the co-transplantation group and only 20% (1 of 5) in the OPC-alone group, while no detection was observed in the control group. Meanwhile, the calretinin^+^ sensory neurons increased in the co-transplanted group, which piqued our interest. However, these increased sensory neurons could not co-localize with GFP, indicating that they may not be derived from grafts, but from their own restoration or the protection of the residual cells by grafts. Moreover, CGRP^+^ cells were increased in transplantation groups, but no significant difference among the three groups. For this, more experiments related to sensory neuron repair will be conducted both in vitro and in vivo. Studies have shown that BDNF can be expressed in primary sensory neurons and participate in regulating painful stimuli and also increase neuronal survival in vitro and in vivo [48, 65–68]. While HUVECs and OLs could secrete various factors, including BDNF [24–28, 68, 69]. In addition, monocarboxylate transporter 1 (MCT1), a lactate transporter, exists in OPCs and HUVECs. When OPCs are damaged during SCI, the reduction of MCT1 also can cause axonal damage and loss of neurons [13, 70, 71]. Consequently, adding exogenous OPCs and HUVECs may play a certain protective role in neuron survival after SCI. In this study, we noticed that HUVECs were unable to be detected 2 weeks after implantation, making it unclear whether these nutritional factors secreted by HUVECs are related to the recovery of sensory function. We speculated that HUVECs, in the early stages of spinal cord injury, may exert a beneficial effect by either secreting soluble factors or cell–cell interactions to improving the microenvironment. There is still much to be investigated regarding motor and sensory functional recovery, including neural circuit reconstruction between grafts and host cells, as well as the role of HUVECs in functional recovery.

In the current study, only female rats were used due to a higher degree of ease of post-injury care and the less aggressive nature. However, we acknowledge that randomly cycled female rats and the uneven levels of hormones such as estrogen may exert a certain impact on the functional recovery of the SCI rats. Future studies using only males or both males and females are warranted to address this issue.

## Conclusions

Here, we have demonstrated that after transplantation into the injured spinal cord, grafts have the capacity for migration, remyelination, and synapse formation and promote motor function recovery. Furthermore, co-transplantation with HUVECs improves sensory function recovery. In this study, we developed a novel combinatory transplantation strategy to enhance the treatment efficacy using hiPSC-OPCs and HUVECs for SCI. Gaining insight into how HUVECs affect the host sensory system would be a new perspective on spinal cord injury.

### Supplementary Information

**Additional file 1. **Supplementary Figure S1, Figure S2, Figure S3, Figure S4, and Table S1.

## Data Availability

All data needed to evaluate the conclusions in the paper are present in the paper and/or the Additional file 1.

## Abbreviations

SCI: Spinal cord injury
iPSCs: Induced pluripotent stem cells
OPCs: Oligodendrocyte progenitor cells
ESCs: Embryonic stem cells
NSCs: Neural stem cells
NPCs: Neural progenitor cells
NG2-OPCs: Neuron-glial antigen 2-expressing OPCs
hiPSC-OPCs: Human iPSC-derived OPCs
OLs: Oligodendrocytes
EDTA: Ethylenediamine tetraacetic acid
PAX6: Paired box 6
OLIG2: Oligodendrocyte lineage transcription factor 2
OCT4: Octamer binding transcription factor 4
O4: Oligodendrocyte 4
MBP: Myelin basic protein
GFAP: Glial fibrillary acidic protein
VEGF: Vascular endothelial growth factor
O.C.T compound: Optimal cutting temperature compound
DPBS: Dulbecco's phosphate-buffered saline
PBS: Phosphate-buffered saline
PBST: Phosphate-buffered saline with 0.3% triton X-100
MEP: Motor evoked potential
SEP: Sensory evoked potential
CM: Conditioned medium
CNS: Central nervous system
GFP: Green fluorescence protein
HUVECs: Human umbilical vein endothelial cells
BBB: Basso–Beattie–Bresnahan
MCT1: Monocarboxylate transporter 1
MVECs: Microvascular endothelial cells
BDNF: Brain-derived neurotrophic factor
IGF-1: Insulin-like growth factor-1
FGF-2: Fibroblast growth factor 2
PDGF: Platelet-derived growth factor
PDGFR-α: Platelet-derived growth factor receptor alpha
NT3: Neurotrophin 3
T3: Triiodo-l-thyronine
HGF: Hepatocyte growth factor
Caspr: Contactin-associated protein
PECAM-1: Platelet endothelial cell adhesion molecule-1
