# Supplementary material for: Grafted human-induced pluripotent stem cells-derived oligodendrocyte progenitor cells combined with human umbilical vein endothelial cells contribute to functional recovery following spinal cord injury
Source: Stem Cell Res Ther. 2024 Feb 7;15:35. doi: 10.1186/s13287-024-03651-1 (PMC10848469; doi:10.1186/s13287-024-03651-1)
Supplement: Supplementary file 1 — Additional file 1. Supplementary Figure S1, Figure S2, Figure S3, Figure S4, and Table S1. [file 13287_2024_3651_MOESM1_ESM.pdf]

Additional file 1 for

Grafted human iPSC-derived oligodendrocyte progenitor cells combined with HUVECs contribute to functional recovery following spinal cord injury

Qian Li, Sumei Liu, Tianqi Zheng, Mo Li, Boling Qi, Liping Zhou, Bochao Liu, Dan Ma, Chao Zhao, Zhiguo Chen*

*Corresponding author. Email: chenzhiguo@gmail.com

This file includes:

Figure S1 to S4

Tables S1

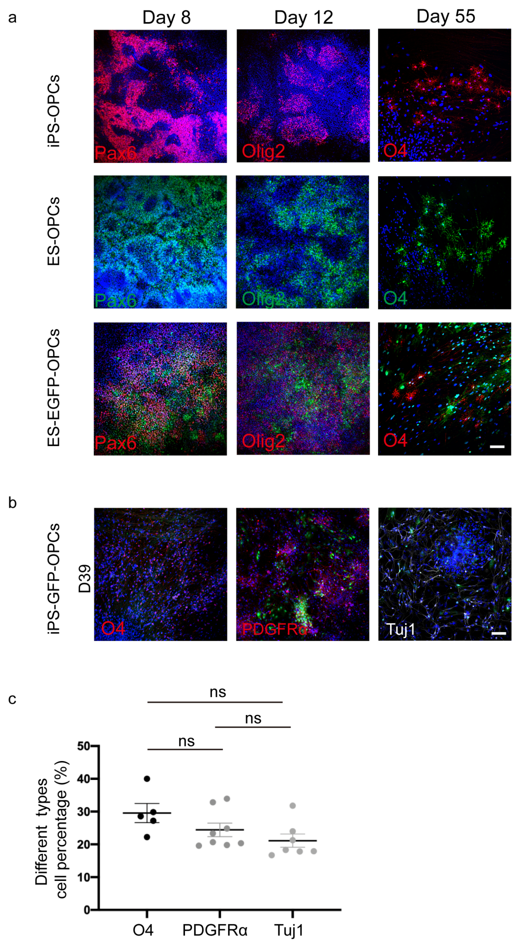

Figure S1 Three cell lines were successfully induced to differentiate into OPCs

a. PAX6, OLIG2, and O4 expression at day 8, day 12, and day 55 of iPS-OPCs (PAX6/OLIG2/O4, red; DAPI, blue), ES-OPCs (PAX6/OLIG2/O4, green; DAPI, blue), and ES-EGFP-OPCs (PAX6/OLIG2/O4, red; DAPI, blue), as shown through immunofluorescence analysis (O4, red; DAPI, blue). Scale bars, 100 μm.

b. Identification of different types of differentiation cells on day 39 (O4/PDGFRα, red; Tuj1, white; DAPI, blue). Scale bars, 100 μm.

c. Different cell proportions before transplantation, the percentage of $O4^{+}$ cells were about 29.6 ± 2.9% of the total cells, $PDGFR\alpha^{+}$ cells were 24.4 ± 2.9%, and $Tuj1^{+}$ cells were around 24.4 ± 3.7%. Data are means ± SEM, ns, no significant; *p<0.05, One-way ANOVA, N=3 independent experiments.

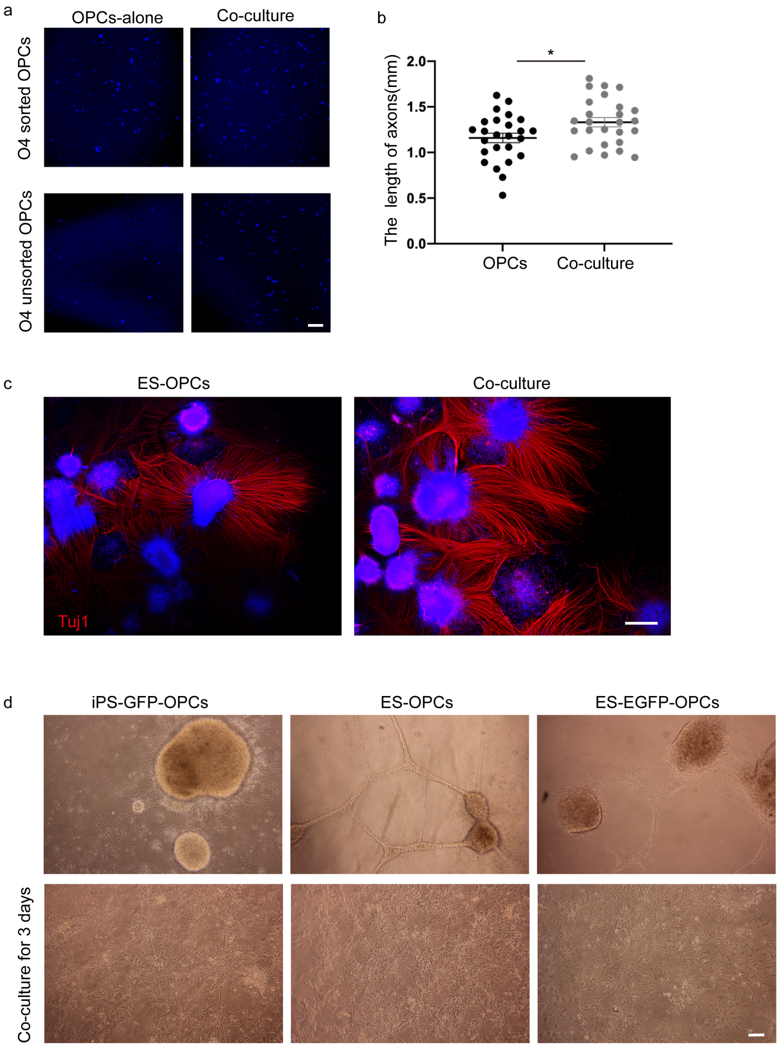

Figure S2 HUVECs promoted OPCs migration, survival, and neuronal axonal growth *in vitro*

a. DAPI staining of migrated cells (DAPI, blue). Scale bars, 100μm.

b. Compared with the control group, the axon length of the co-cultured group was significantly increased. Data are means ± SEM. *$p<0.05$, t test, N=3 independent experiments.

c. Immunofluorescence staining of axons extension of ES-OPCs cultured and co-cultured with HUVECs after three days (Tuj1, red; DAPI, blue). Scale bars, 750 μm.

d. OPCs differentiated from iPS-GFP/ES/ES-EGFP were digested on day 45 and then cultured alone and co-cultured with HUVECs for 3 days. The ability of axon extension, cell migration, and survival was better in the co-culture group. Scale bars, 100μm. N=3 independent experiments.

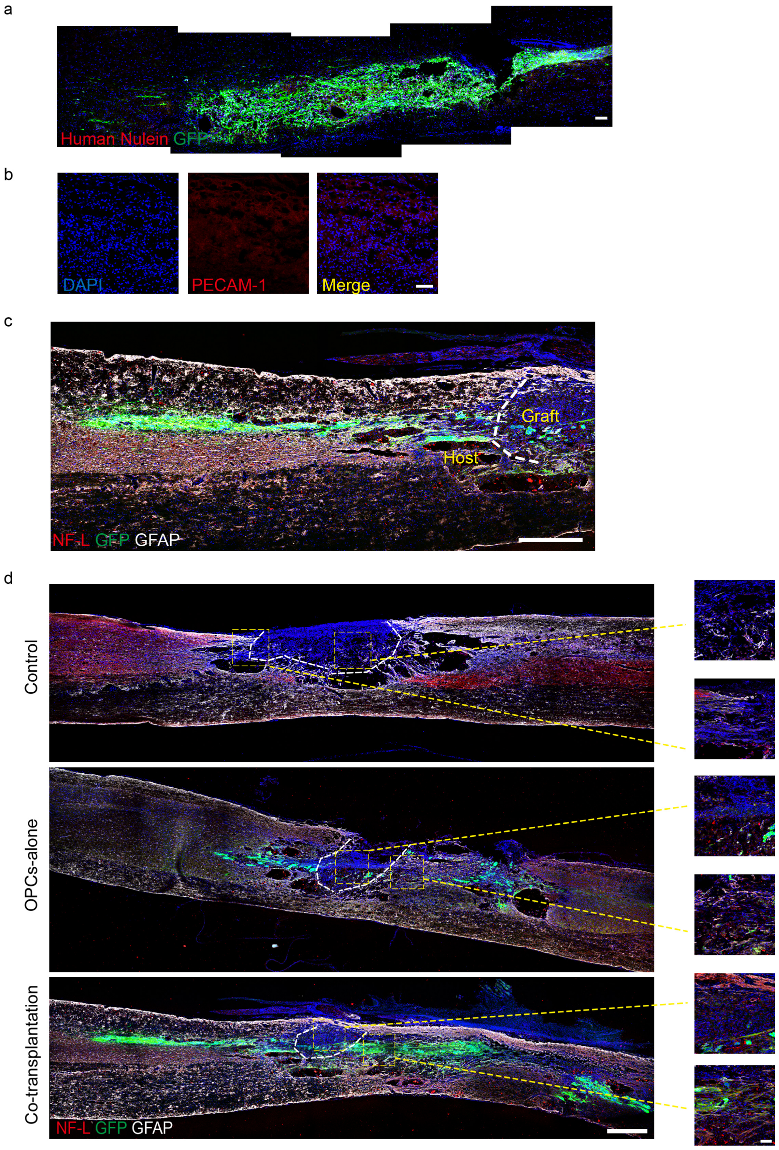

Figure S3 Survival, migration of grafts in the SCI model

a. iPSCs-OPCs survived 2 weeks post-transplantation in vivo. Scale bar, 100 μm.

b. HUVECs could not be detected after 2 weeks implanted. Scale bar, 75 μm.

c. Transplanted (GFP^{+}) cells survived, and migrated across the lesion border (NF-L, red; GFP, green; GFAP, white). Scale bar, 500 μm.

d. Immunofluorescence staining of all groups 8 weeks post-transplantation showed that the tissue defect, and the GFP^{+}, $NF\text{-}L^{+}$ cells in the injury area, the high-magnification view of the regions showed on the right side (GFP, green; NF-L, red; GFAP, White). Scale bar, left panel 500 μm, right panel 75 μm.

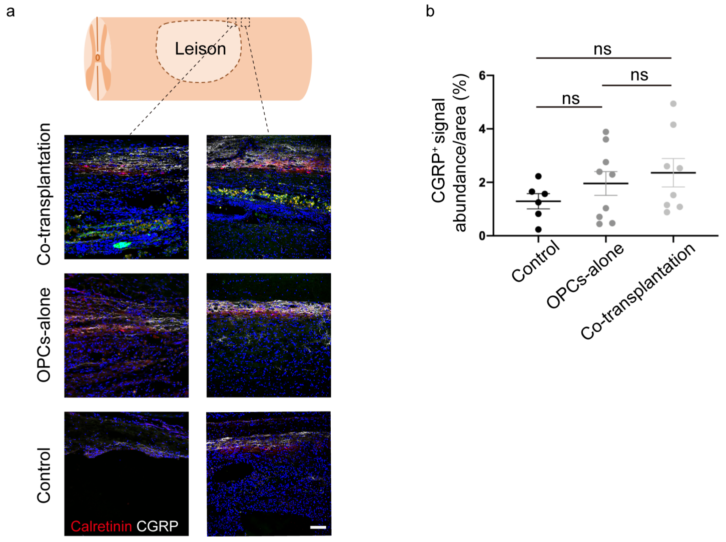

Figure S4 CGRP expression in three groups 2 months post-transplantation

a. Immunofluorescence staining of CGRP in injury border of all groups. Scale bar, 100 μm

b. Compared with the CGRP pixel abundance of the control group in and around the lesion site. Data are means ± SEM. ns, no significant; *p<0.05, One-way ANOVA. Scale bar, 75 μm. n=5 rats in each group.

Table S1

Antibody list

Primary antibody	Host	Dilution	Vendor	60
Caspr	rabbit	1:500	abcam	
GFAP	rabbit	1:500	Abcam	
GFP	Goat	1:1000	Invitrogen	
Human Nuclei	mouse	1:500	Millipore	
Stem121	mouse	1:500	cellartis	
MBP	rat	1:200	abcam	
NF-L	chicken	1:200	abcam	
NF200	mouse	1:300	Millipore	
OCT4	mouse	1:200	Santa cruz	
O4	mouse	1:1000	Millipore	
OLIG2	rabbit	1:500	Millipore	
PAX6	rabbit	1:200	ebioscience	
PECAM-1	mouse	1:400	santa cruz	
SYNAPSIN	mouse	1:500	Millipore	
TUJ-1	mouse	1:500	Millipore	
TUJ-1	rabbit	1:1000	abcam	
